# Enzymatic Defense Response of Apple Aphid *Aphis pomi* to Increased Temperature

**DOI:** 10.3390/insects11070436

**Published:** 2020-07-11

**Authors:** Jan Dampc, Monika Kula-Maximenko, Mateusz Molon, Roma Durak

**Affiliations:** 1 Department of Experimental Biology and Chemistry, University of Rzeszów, Pigonia 1, 35-310 Rzeszów, Poland; jdampc@ur.edu.pl; 2 The Franciszek Górski Institute of Plant Physiology, Polish Academy of Sciences, Niezapominajek 21, 30-239 Kraków, Poland; kula.monika@gmail.com; 3 Department of Biochemistry and Cell Biology, University of Rzeszow, Zelwerowicza 4, 35-601 Rzeszow, Poland; mateuszmolon@univ.rzeszow.pl

**Keywords:** aphids, environmental stress, enzymatic markers

## Abstract

Climate change, and in particular the increase in temperature we are currently observing, can affect herbivorous insects. Aphids, as poikilothermic organisms, are directly exposed to temperature increases that influence their metabolism. Heat stress causes disturbances between the generations and the neutralization of reactive oxygen species (ROS). The aim of this work is focused on explaining how the aphid, using the example of *Aphis pomi*, responds to abiotic stress caused by temperature increase. The experiment was carried out under controlled conditions at three temperatures: 20, 25, and 28 °C. In the first stage, changes in the activity of enzymatic markers (superoxide dismutase (SOD), catalase (CAT), glutathione S-transferase (GST), β-glucosidase, polyphenol oxidase (PPO), and peroxidase (POD)) were determined in aphid tissues, at each temperature. In the second stage, microcalorimetry monitored changes in heat emitted by aphids, at each temperature. Our results showed that *A. pomi* defense responses varied depending on temperature and were highest at 28 °C. The flexible activity of enzymes and increase in the metabolic rate played the role of adaptive mechanisms and ran more effectively at higher temperatures. The *A. pomi* thus protected itself against ROS excessive induction and the aphids were able to respond quickly to environmental stress.

## 1. Introduction

We are currently observing climate changes that may influence herbivorous insects. Temperature is an important factor that affects distribution and life cycles in insects. Next to the photoperiod, this is one of the most important environmental factors that aphids react to [1,2]. Aphids, like other insects, are poikilothermic organisms; they reproduce only within a certain temperature range. These insects have narrow thermal tolerance, and their rate of development depends on temperature. It has been demonstrated that aphids respond to a 2 °C increase of temperature with one to five additional cycles per season [3]. Temperature also affects the species’ phenology, spread range, life cycles, and migration times [1,2,4,5]. Aphids have a number of features desirable for model organisms, such as: rapid development of generations, high number of generations, high fertility of females, and telescopic generations that allow shortening of development [4,6]. These features allow aphids to react quickly to climate change. Sensitivity to the environment makes aphids good indicators of the impact of climate change on organisms [2]. The increase in temperature not only influences the biology of insects but also affects them on a cellular level and their metabolism. This is why the issue of aphids’ defense responses to high temperatures is of great interest.

High temperature at cellular level can cause mitochondrial dysfunction, affecting oxidative phosphorylation and cellular respiration [7]. The temperature above the thermal optimum is perceived by the body as heat stress and leads to imbalance between the production of reactive oxygen species (ROS) and antioxidant processes [8,9]. The rapid increase in ROS levels in tissues causes oxidative stress (OS) and damage to DNA, lipids, or proteins [10,11]. Generating ROS in the body occurs naturally as a product of many metabolic processes. For example, ROS can be produced due to the autooxidation of some particles, oxidoreductase activity, and as a byproduct in the electron transport chain [12]. During the incomplete reduction of O_2_ in the process of cellular respiration, the superoxide anion radical (O_2_^•−^), hydroxyl radical (OH^•^), and hydrogen peroxide (H_2_O_2_) are generated. Aphids are also exposed to ROS from exogenous sources. ROS can be produced by plants as protection against phytophagous insects [13]. In addition, plant tissue damage and saliva produced by aphids can induce ROS generation in plant cells [14].

To prevent OS induction, aphids have developed systems responsible for neutralizing ROS. The main enzymes responsible for scavenging them are antioxidant enzymes such as superoxide dismutase (SOD) and catalase (CAT) [13,14]. In the aphid’s organism, xenobiotics are metabolized to less toxic compounds [15], by detoxification enzymes such as β-glucosidase and S-glutathione transferase (GST) [16], oxidoreductive peroxidase (POD), and polyphenol oxidase (PPO) [17]. GST with the participation of glutathione neutralizes allelochemicals involved in the generation of ROS in the insect’s body [18]. PPO and POD oxidoreductases are involved in the metabolism of plant phenolic compounds. They play a key role in the aphid’s digestion process, as the presence of these enzymes allows the aphid to neutralize a wide range of phenolic compounds [17]. Insects maintain homeostasis in the body by neutralizing ROS and xenobiotics by activating various metabolic pathways, which help protect cellular components from oxidative stress [19]. Change in the activity of enzymatic markers in the tissues of aphids, is proof of the enzymatic defense response of aphids. Fast coordinated interaction of antioxidative (SOD, CAT), oxidoreductive (PPO, POD), and detoxification (GST, β-glucosidase) enzymes enables the neutralization of ROS and harmful metabolites during temperature stress.

*Aphis pomi* (De Geer, 1773) (Hemiptera: Aphidoidea), is a holocyclic and monoecious species. This oligophagous insect feeds on trees and shrubs of the Rosaceae family such as apple, pear, hawthorn, and quince. The species often forms large, strong honey-dewing colonies, which cause leaves to curl, and impair the development of young shoots. *A. pomi* is a serious pest of economically important trees grown in commercial orchards, and causes damage or a decrease in the aesthetic value of many decorative plants. It has been shown that this species can develop an anholocyclic life cycle, which is influenced by abiotic factors such as temperature and photoperiod, or biotic factors such as the quality of the host plant [20,21].

The aim of the study was to determine the effect of temperature increase on aphids, with the example of *A. pomi*. We decided to verify two hypotheses: (1) An increase in temperature increases the enzymatic defense response of aphids. (2) An increase in temperature increases the metabolic activity of *A. pomi.*

## 2. Materials and Methods

### 2.1. Aphids

The *A. pomi* aphids were taken from the wild in spring and kept under controlled conditions in a climate chamber (MLR-351H; Sanyo Corp., Osaka, Japan) at the University of Rzeszow for three generations. Aphids were reared at 20 ± 1 °C, 60 ± 5% humidity, and 16L: 8D photoperiod. Wingless females from the third generation were used for each experiment.

### 2.2. Host Plants

The host plants were 2-year-old seedlings of *Chaenomeles japonica*. Plants were in pots: 30 cm × 30 cm × 30 cm and were free of pathogens. Before the start of the experiment, they were kept at 20 °C for 2 weeks. *Ch. japonica* is a very popular decorative plant belonging to the Rosaceae family, on which aphids develop and reproduce prolifically. In all experiments we used the same host plant, grown under the same conditions, at the same development phase, in order to show the effect of temperature on the enzymatic activity of aphids, and to eliminate the influence of other factors. For testing, we chose the temperatures 20, 25, and 28 °C, which are common temperatures in temperate climates and do not cause heat shock in many plants, and are within the range of quince’s heat tolerance. For aphids, the optimal temperature for development is about 20–25 °C, while temperatures close to 30 °C are lethal.

### 2.3. Effect of Temperature on Enzymatic Activity in Aphid Tissues

The experiments were carried out at the three temperatures of 20, 25 and 28 °C in a climate chamber (MLR-351H; Sanyo Corp., Japan) with a constant humidity of 60 ± 5% and a 16L: 8D photoperiod. We applied 30 aphids to each seedling *Ch. japonica*, which lived on the separated host plants for 24, 48, 72, 96, and 336 h (2 weeks). The control consisted of aphids collected directly from the starting culture at 20 °C. The experiment was performed in 3 replications. The samples (each sample had 30 aphids) were then collected at each time period and frozen in liquid nitrogen. Samples for analysis were kept at −85 °C (Deep freezer VXS 490, Thermo Scientific, Berlin, Germany).

#### 2.3.1. Homogenization of Aphids

Aphids (30 individuals) were placed in a phosphate buffer (0.1 M, pH 7.0) then homogenized at 0 °C. The homogenate was centrifuged (Eppendorf Centrifuge 5810 R, Eppendorf, Hamburg, Germany) at 4 °C. The supernatant was used for subsequent determinations of enzymatic activity. All biochemical assays were performed in three independent biological replicates (n = 3) for each enzyme, at three different temperatures, and for each time period.

#### 2.3.2. Superoxide Dismutase (SOD) Activities

The SOD activity was determined using a standard method [12]. Composition of reaction mixture: phosphate buffer (0.2 M pH 7.8), homogenate, NBT (0.25 mM) (Sigma-Aldrich, Saint-Louis, MO, USA) in phosphate buffer (0.2 M pH 7.8), and xanthine (Sigma-Aldrich, Saint-Louis, MO, USA) solution (0.25 mM). The mixture was incubated for 20 min and then absorbance measured at 560 nm (TECAN Infinite 200 microplate reader, Grödig, Austria. The activity was the amount of enzyme needed to inhibit the reaction of producing peroxide anion by half (U × min^−1^ × mg protein^−1^).

#### 2.3.3. Catalase Activity (CAT)

CAT activity was measured using the method described by Aebi [22] with a slight modification. We added 30 mM H_2_O_2_ (Sigma-Aldrich, Saint-Louis, MO, USA) to the aphid homogenate, and the disappearance of H_2_O_2_ was measured at 240 nm during 1 min (Carry 50). Catalase activity was expressed as µmol H_2_O_2_ × min^−1^ × mg protein^−1^.

#### 2.3.4. β-Glucosidase Activity

The activity of β-glucosidase was determined by the hydrolysis reaction of *p*-nitrophenyl-β-D-glucopyranoside with β-glucosidase [23]. The reaction mixture consisted of: 50 mM solution of *p*-nitrophenyl-β-D-glucopyranoside (Sigma-Aldrich, Saint-Louis, MO, USA), 0.2 M phosphate buffer (pH 5.8), and extract. The whole was incubated for 30 min (25 °C), and then 2% Na_2_CO_3_ was added. The absorbance (TECAN Infinite 200 microplate reader) was measured at 400 nm. Activity was expressed as nmol × min^−1^ × mg protein^−1^.

#### 2.3.5. Glutathione S-Transferase (GST) Activity

The activity of GST was based on the reaction of 1-chloro-2,4-dinitrobenzene (CDNB) (Sigma-Aldrich, Saint-Louis, MO, USA) with reduced glutathione (GSH) (Sigma-Aldrich, Saint-Louis, MO, USA) [24]. Composition of reaction mixture: extract and 20 mM GSH. The mixture was incubated for 10 min at 30 °C, followed by the addition of 100 mM CDNB (Sigma-Aldrich, Saint-Louis, MO, USA) and was incubated for a further 5 min at 30 °C. Absorbance was measured (TECAN Infinite 200 microplate reader) at 340 nm. Activity was expressed as nmol × min^−1^ × mg protein^−1^.

#### 2.3.6. Polyphenol Oxidase (PPO) Activity

PPO activities were determined using the method described by Miles [25] with a modification [26]. The reaction mixture consisted of 0.2 M phosphate buffer (pH 7.4), extract, and 10 mM catechol solution (Sigma-Aldrich, Saint-Louis, MO, USA). The mixture was incubated at 30 °C for 30 min. The measured absorbance was compared with the control, which contained 0.2 M phosphate buffer (pH 7.4) instead of the extract. Measurements were taken (TECAN Infinite 200 microplate reader) at a wavelength of 460 nm. Activity was shown as: ΔA460 × min^−1^ × mg protein^−1^.

#### 2.3.7. Peroxidase (POD) Activity

POD activity was determined using the method described by [27]. Reaction mixture: 0.1 M phosphate buffer (pH 7.0), extract, distilled water, 0.2 M pyrogallol, and 3% H_2_O. The whole was incubated at 30 °C for 25 min, and then a 25% trichloroacetic acid (TCA) (Sigma-Aldrich, Saint-Louis, MO, USA) solution was added. Absorbance was measured at 430 nm (TECAN Infinite 200) against a blank test in which 0.1 M phosphate buffer (pH 7.0) was added instead of the extract. The enzyme activity was: µmol × min^−1^ × mg protein^−1^.

#### 2.3.8. Protein Content in Aphid Tissue Homogenates

Protein content was determined using a standard method [28]. The calibration curve was determined on the core albumin solution (Sigma-Aldrich, Saint-Louis, MO, USA). The protein content was expressed as mg/mL.

### 2.4. Effect of Temperature on Metabolic Activity of Aphids Using Isothermal Calorimetry

Metabolic activity of *A. pomi* was measured at 20, 25, and 28 °C using a TAM III isothermal calorimeter equipped with TAM Assistant Software (TA Instruments, Lindon, UT, USA). Twenty aphids on a young leaf were placed into 4.0 mL calorimetric ampoules with 0.25 μL of water. After 30 min, which is needed to balance the temperature, thermal power curves were recorded over 24 h.

Each measured thermal power curve combined the thermal power curve from the leaf and aphids. In order to distinguish the thermal power curve from aphids, reference thermal power curves for the leaf itself were taken before each measurement and later deducted from the thermal power curve. Specific thermal power curves [mW × g^−1^] were obtained by standardizing thermal power curves from aphids to their mass. Metabolic activity of *A. pomi* in the form of heat energy [J × g^−1^] was calculated as the area under the specific thermal power curve. Presented data show averaged values from 12 independent repetitions for each specimen.

### 2.5. Statistical Analyses

Data were tested for normality and homogeneity of variances. Two-way analysis of variance (ANOVA), with temperature and time as fixed factors, was used to test for differences between the average enzymatic activities, and post hoc comparisons were undertaken using the Duncan multiple test range. Each experiment was performed in 3 replications. We compared averaged values of enzymatic activities (dependent variables, response) depending on the two explanatory variables (factors): temperature (range tested 20, 25, and 28 °C) and time (0, 24, 48, 72, 96, and 336 h). We compared 48 samples to examine the activity of each enzyme. Analyses were performed separately for each enzyme. All data are presented as means with standard error values (mean ± SE). The impact of the temperatures on the metabolic activity of aphids was evaluated by the one-way ANOVA multiple range test (*p* < 0.05). The results were expressed as mean ± SE and were separated using post hoc Tukey’s HSD test. Statistical analyses were performed using the statistical program Statistica (data analysis software system), version 13 (TIBCO Software Inc., 2017, http://statistica.io).

## 3. Results

### 3.1. Effects of Temperature on Aphid Enzyme Activity

#### 3.1.1. Superoxide Dismutase (SOD) and Catalase (CAT) Activity

**Figure 1:**
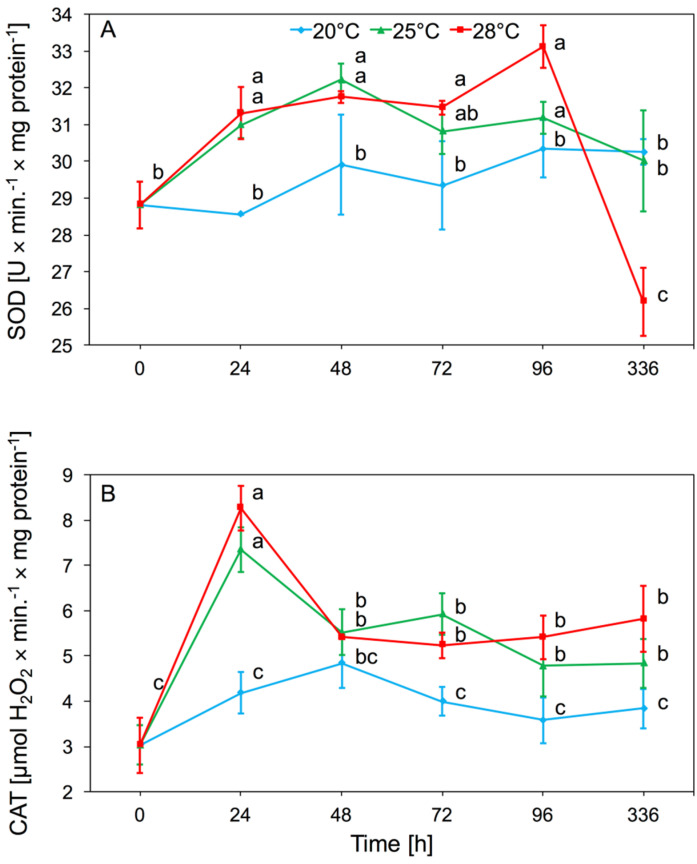
Activity change of superoxide dismutase (SOD) (**A**) and catalase (CAT) (**B**) in the tissues of *Aphis pomi* at temperature 20, 25, and 28 °C (mean ± SE; control = aphids at 20 °C). Values not followed by the same letter are significantly different at the level of *p* < 0.05 (Duncan multiple range test).

Analyses of the enzymatic activity in *A. pomi* tissues after the onset of foraging showed an increase in SOD activity at 25 and 28 °C. Temperature had a significant effect on SOD activity in aphid tissues (two-way ANOVA F_(2,32)_ = 6.01, *p* < 0.001), as did time (two-way ANOVA F_(4,32)_ = 7.21, *p* < 0.001). There was significant interaction between temperature and time in SOD activity (two-way ANOVA F_(8,32)_ = 5.74, *p* < 0.001). We observed a significant increase in activity of SOD, occurring after 24 h and 48 h at 25 and 28 °C. At 20 °C enzyme activity was similar to the control. The increased level of SOD activity persisted until the 96th hour of the experiment and returned to control level after two weeks. The lowest values of SOD activity were observed at 20 °C; at this temperature there were no significant differences in activity compared to the control (Figure 1A).

The main effect of temperature on CAT activity in aphid tissue was significant (two-way ANOVA F_(2,32)_ = 69.06, *p* < 0.001). Moreover, time had a significant effect on enzyme activity (two-way ANOVA F_(4,32)_ = 17.25, *p* < 0.001). There was also significant interaction between temperature and time in CAT activity (two-way ANOVA F_(8,32)_ = 5.41, *p* < 0.001). Increased CAT activity in *A. pomi* tissues was observed on the first day that aphids were kept at 25 and 28 °C. Activity at these temperatures after 48 h decreased gradually, but up to 96 h was higher in comparison to activity at 20 °C. After two weeks of the experiment, CAT activity at 25 °C and 28 °C was still higher than activity at 20 °C. There was no significant increase in CAT activity in aphids reared at 20 °C (Figure 1B).

#### 3.1.2. β-Glucosidase and S-Glutathione Transferase (GST)

**Figure 2:**
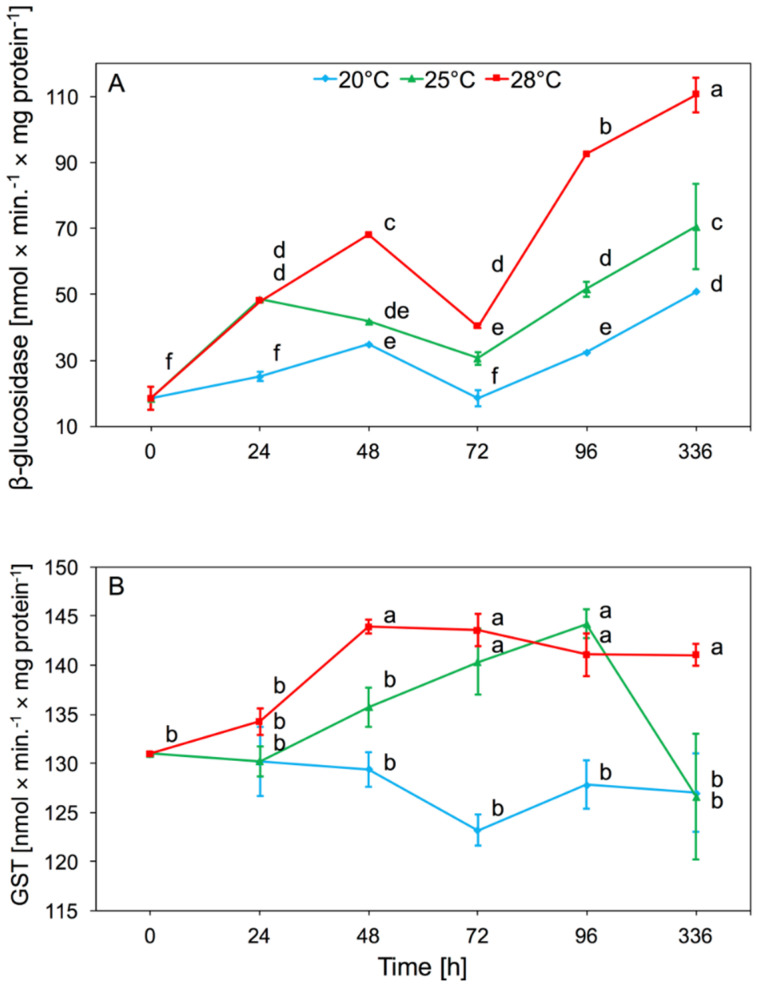
Activity change of β-glucosidase (**A**) and s-glutathione transferase (GST) (**B**) in the tissues of *A. pomi* at temperature 20, 25, and 28 °C (mean ± SE; control = aphids at 20 °C). Values not followed by the same letter are significantly different at the level of *p* < 0.05 (Duncan multiple range test).

Temperature had a significant effect on β-glucosidase activity in aphid tissues (two-way ANOVA F_(2,32)_ = 223.4, *p* < 0.001), as did time (two-way ANOVA F_(4,32)_ = 120.7, *p* < 0.001). There was also significant interaction between temperature and time in β-glucosidase activity (two-way ANOVA F_(8,32)_ = 14.02, *p* < 0.001). We observed a significant increase in activity after 24 h and 48 h in aphid tissues, both at 25 and 28 °C. The increase in activity was maintained until the end of the experiment. Only a slight increase in the activity of this enzyme at 20 °C was observed. We noted that along with the increase in temperature, the enzymatic activity of β-glucosidase was higher. During the experiment, the highest β-glucosidase activity was observed in aphids at 28 °C. The β-glucosidase activity increased significantly in the tissues of aphids which were reared under elevated temperature for two weeks (Figure 2A).

The GST activity found in aphid tissues differed significantly depending on temperature (two-way ANOVA F_(2,32)_ = 37.19, *p* < 0.001) and time (two-way ANOVA F_(4,32)_ = 4.08, *p* < 0.01). We found significant interaction between temperature and time in GST activity (two-way ANOVA F_(8,32)_ = 3.05, *p* < 0.01). At 20 °C, no significant change in the activity of this enzyme was observed relative to the control. The increase in GST activity was observed from 48 h at 28 °C, and after 96 h at 25 °C. After two weeks the level of activity decreased and was comparable to the aphids at 20 and 25 °C, while at 28 °C it was still higher than at 20 °C (Figure 2B).

#### 3.1.3. Polyphenol Oxidase (PPO) and Peroxidase (POD)

**Figure 3:**
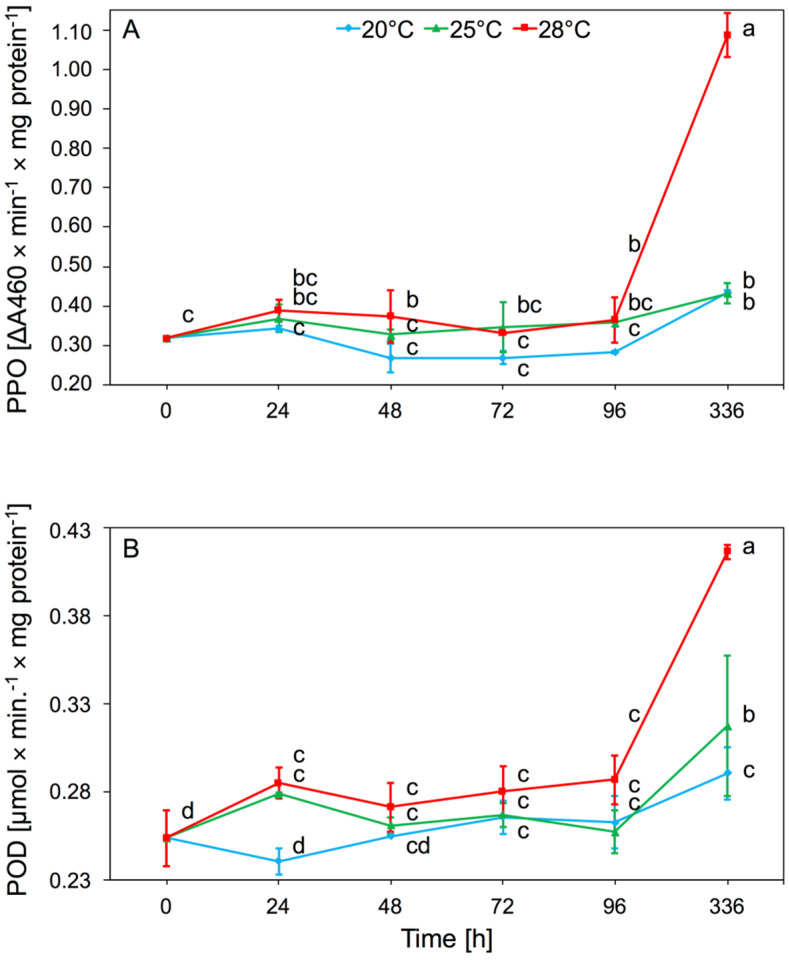
Activity change of polyphenol oxidase (PPO) (**A**) and peroxidase (POD) (**B**) in the tissues of *A. pomi* at temperature 20, 25, and 28 °C (mean ± SE; control = aphids at 20 °C). Values not followed by the same letter are significantly different at the level of *p* < 0.05 (Duncan multiple range test).

The main effect of temperature on PPO activity was significant (two-way ANOVA F_(2,32)_ = 80.53, *p* < 0.001), but time also had an effect (two-way ANOVA F_(4,32)_ = 88.47, *p* < 0.001). There was significant interaction between temperature and time in PPO activity (two-way ANOVA F_(8,32)_ = 35.31, *p* < 0.001). The increase in activity occurred after 24 h but was not statistically significant. After 48 h at 28 °C, there was a significant increase in activity of this enzyme in aphid tissues, relative to 20 °C. The highest value of PPO activity was observed after 2 weeks at 28 °C. The highest PPO activity was observed at 28 °C (Figure 3A).

Temperature significantly affected POD activity present in aphid tissues (two-way ANOVA F_(2,32)_ = 20.84, *p* < 0.001). In addition, the time of infestation by aphids had an effect (two-way ANOVA F_(4,32)_ = 27.98, *p* < 0.001). There was also significant interaction between temperature and time in POD activity (two-way ANOVA F_(8,32)_ = 4.64, *p* < 0.001). The increase in POD activity was observed after 24 h of keeping aphids at 25 and 28 °C, and then remained at this level up to 96 h. Significant increase in activity of POD was observed after 2 weeks at 28 °C (Figure 3B).

### 3.2. Determination of the Metabolic Activity of Aphids

**Figure 4:**
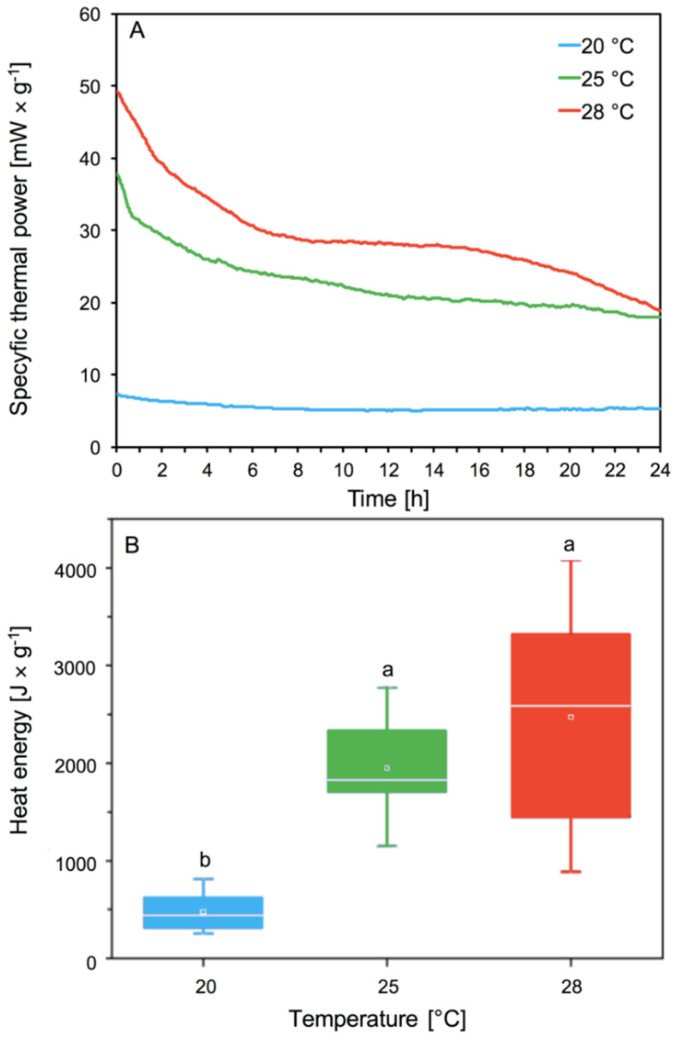
Specific thermal power curves [mW × g^−1^] (**A**) and heat energy (mean ± SE) [J × g^−1^] (**B**) of *A. pomi* at temperature 20, 25, and 28 °C. Values not followed by the same letter are significantly different at the level of *p* < 0.05 (Tukey test).

Our analyses of specific thermal power curves [mW × g^−1^] showed the significant effect of temperature on the metabolic activity of the aphids. Aphids’ thermal power increases with the increase in temperature (Figure 4A). Differences were observed in the intensity and shape of the curves. We observed the lowest thermal power emission for aphids living at 20 °C in comparison to higher temperatures (*p* < 0.05) (Figure 4A). The highest thermal power emission was observed at 28 °C (Figure 4A). The aphids at 28 °C emitted the most thermal power, and consumed oxygen much faster than at lower temperatures. At the lowest temperature of 20 °C, the aphids did not lose much energy and did not consume oxygen so quickly. Aphids reared at 25 °C lost less thermal energy and at the same time consumed less oxygen than aphids living at 28 °C (Figure 4A).

Heat energy signifies the metabolic activity of aphids depending on their temperature. The heat energy produced by aphids in all conditions increased with increasing temperature from about 500 [J × mg ^−1^] at 20 °C to about 2500 [J × mg ^−1^] at 28 °C (Figure 4B). Aphids living at 20 °C showed the lowest metabolic activity, while aphids at 25 and 28 °C had statistically significant higher metabolic activity (Figure 4B). No significant differences in emission of heat energy were observed between 25 and 28 °C. Higher aphid metabolic activity resulted in greater and faster oxygen consumption.

## 4. Discussion

One of the effects of thermal stress is excessive ROS generation which causes oxidative damage [11]. Correctly functioning defense systems allow the body to maintain undisturbed physiological and metabolic processes. The key role in the defense of the aphid organism is that of their enzymatic defense. Antioxidative, detoxifying, and oxidoreductive enzymes are the most significant enzymes involved in the neutralization of ROS and xenobiotics [29]. Our research showed how activity of these enzymes in *A. pomi* depends on temperature.

The first stage of the aphid’s response to oxidative stress is an increase in SOD activity. The activity of this enzyme is dependent on the temperature and gradually increases in aphid tissues at 25 and 28 °C (Figure 1A). At 20 °C, no significant changes in activity were observed. SOD is involved in the transformation of toxic induction of superoxide dismutase activity that catalyzes O_2_^•−^ to O_2_ and H_2_O_2_ dismutations. CAT catalyzes the H_2_O_2_ reduction reaction to water. An increase in CAT activity was observed as early as 24 h after starting the experiment; this level gradually decreased in subsequent days at each temperature, but the highest activity of this enzyme that we observed during the whole experiment was at 28 °C (Figure 1B). It was interesting that after a period of 2 weeks, the activity of the enzyme in the aphids was still higher at 25 and 28 °C than at 20 °C. Our research showed that the activity of SOD and CAT enzymes in *A. pomi* tissues increased at higher temperatures and achieved the highest activity at 28 °C. This showed that exceeding the optimum temperature for the species caused a quick defensive response from the first day of the stressor’s action. The activity of antioxidant enzymes in aphid tissues is also associated with the host plant spectrum. It was shown that SOD and CAT activity in the tissues of *Rhopalosiphum padi* and *Sitobion avenae* was associated with a change in the host plant and higher activity was observed in polyphagous aphids [14]. *A. pomi* as an oligophagous species was characterized by higher SOD activity than *Cinara tujafilina*, which also has a broad spectrum of host plants [30]. Differences in aphids’ SOD activity probably resulted from the various chemical compositions of the host plants, which affected aphid response. Quercetin and phenylpropenoic acids influenced SOD activity, which correlated with the concentration of these compounds in the aphids’ diet [31]. A change in the activity of antioxidant enzymes was also observed, as a result of adaptation to various host plants, in *Myzus persicae*, which is a highly polyphagous species [32]. Antioxidative enzymes also played a significant role in insects with chewing mouths, in which an increase in temperature affected the increase in SOD and CAT activity [33]. Studies on *Mythimna separata* showed that an increase in temperatures above the optimum temperature causes an increase in SOD and CAT activity [34]. In the aphid parasite, *Aphidius gifuensis*, SOD, CAT, GST, and POD activity also significantly increased in temperatures above 30 °C [35].

GST and β-glucosidase activity increased in *A. pomi* after 24 h of the experiment (Figure 2A,B). The GST induction suggested that the increase in temperature caused the accumulation of toxic lipid peroxidation products and GST was involved in their inactivation [11,36], which was observed in *A. pomi* from 48 h. An increase in GST activity was also observed as a result of a change in the host plant in *R. padi* and *S. avenae* cereal aphids [18], and *C. tujafilina* [37], which were treated with secondary plant metabolites [18]. The increase in GST activity is an essential indicator of the adaptation of insects to changes in xenobiotics, including phenols, present in plants [17]. Changes in β-glucosidase and GST activity were noted in migration from primary to secondary host of winged *R. padi* females [38] and after the change of host plant in *C. tujafilina* [37]. β-glucosidase activity is strongly associated with the chemical composition of the host plant. An increase in temperature in the range from 10 to 35 °C caused an increase in β-glucosidase activity in *Eurygaster maura* [39], while an increase in temperature above 30 °C in *A. gifuensis* caused an increase in GST activity [35]. β-glucosidase is associated with carbohydrate metabolism, the activity strongly linked with the composition of the sap in host plant, particularly sucrose concentration [37]. β-glucosidase hydrolyzes o-glycosyl bonds carbohydrates such as sucrose [39]. Temperature affects the metabolism of sugars in the plant; an increase in temperature can cause the breakdown of starch [40,41]. The increase in β-glucosidase activity in *A. pomi* tissues during the increase of temperature was probably caused by changes in the carbohydrate metabolism in the host plant. An increase in temperature can cause changes in the chemical composition of phloem sap, especially a decrease in the level of glucose, resulting in a change of place and the start of feeding of aphids in mesophyll cells. Aphids take up other sugars, such as sucrose, and dehydrolyze them to glucose and fructose using β-glucosidase. This could explain the increase in activity of this enzyme in *A. pomi,* especially when temperatures increased to 28 °C, and the aphids took more sucrose from the plant tissue. Aphids may take up more sucrose due to environmental stress, as phloem probing phases are disturbed [42].

Redox enzymes are catalysts for the redox reaction that converts secondary phenolic plant metabolites into less toxic compounds. PPO catalyzes the hydroxylation reactions of monophenols and the further oxidation of these compounds to quinones and non-toxic melanin pigments. With the participation of hydrogen peroxide, POD oxidizes phenols and other aromatic compounds. Both enzymes from this group play a major role in the oxidation of secondary metabolites, which greatly facilitates the absorption of food by insects [43,44]. Our results showed that the activity of POD and PPO in *A. pomi* tissues increased at 25 and 28 °C, and the biggest increase in activity of these enzymes was observed at 28 °C during all experiments (Figure 3A,B). The above studies showed that the activity of these enzymes mainly depended on the biochemistry of the host. Our research showed that long-term thermal stress, acting on a plant infected by aphids, caused an increase in the amount of phenolic compounds contained in plant tissues. Changes in the metabolism of phenolic compounds in plants cause an increase in the number of formed quinones, which are more toxic to aphids [45]. These changes may explain the increase in the activity of these enzymes after 2 weeks of aphid feeding. Aphids take quinones from the plant and neutralize them with PPO and POD to form melanin pigments. This process was particularly evident at 28 °C. This suggests that prolonged high temperatures affect aphid response which depends on temperature. The aphids’ defense response to temperature increase also depends on the duration of the stressor. Aphid defense responses may be visible as early as the first day after exposure to high temperatures, but the prolonged action of the stress factor (two weeks) causes the second stage of defense responses and a re-increase in enzyme activity. An increase in temperature in the range of 25–35 °C in *Propylaea japonica* did not show a significant increase in POD activity [11]. Studies of POD activity in *M. separata* tissues subjected to thermal stress showed that higher POD activity was observed after 1 h and 4 h exposure to stress factors, than after 7 h exposure [34]. An increase in the activity of these enzymes was observed in *S. avenae* after an artificial diet containing gramine [46]. The increase in the activity of these enzymes was a mechanism for aphids to adapt to adverse conditions. An increase in oxidoreductase activity was observed as a result of a change to the host plant of *C. tujafilina* [37].

High temperatures may also have an effect on host plants, including not only loss of water and disappearance of pigmentation, but also disturbance in the process of photosynthesis, and excessive production of ROS [40,41,47]. An increase of temperature in this range resulted in a weak, not statistically significant, increase in the activity of enzymes. An increase in temperature also affected the response of the plant infected by *A. pomi* and we observed that the effectiveness of the plant’s defense response depended on the temperature. Our research showed that the enzymatic defense in aphids increased with increasing temperature, while the activity of these enzymes in host plant tissues showed the opposite trend [48].

The metabolism of living organisms can be measured as the rate of heat emission, CO_2_ production, and O_2_ consumption [49]. Our analyses showed that an increase in temperature from 20 to 25 and 28 °C caused an increase in the specific thermal power of aphids, most notably at 28 °C (Figure 4). Earlier studies indicate that aphids, like other insects, have shorter life expectancy and reduced survival at higher temperatures [50,51,52]. Our research showed that an increase in temperature to 28 °C increases heat energy in *A. pomi* fivefold. Higher levels of metabolism can cause shortened aphid longevity. Aphids in higher temperatures with higher heat flow due to a higher level of metabolism live shorter lives. Our previous research showed the effect of temperature increase on the life cycle of *A. pomi*. We showed that an increase in temperature to 28 °C shortened the longevity of *A. pomi*, reduced the pre-reproduction phase and reproduction phases and also reduced the fecundity of females, and population demographic parameters. The increase of temperature up to 28 °C caused a decrease in survival of *A. pomi,* compared to aphids reared at 20 and 25 °C [48]. The increase in the *A. pomi* metabolic rate observed with increasing temperature, probably also causes changes in feeding time, rate and way of feeding, because aphids take food not only from phloem sap but also from mesophyll tissue. Research based on *Homalodisca vitripennis* confirmed that feeding intensity is dependent on temperature. The feeding intensity increases with rising temperature but environmental temperature below the feeding threshold was a key limiting factor for adult feeding activity [53]. Environmental stress can affect aphids, directly and indirectly, by affecting the host. A relatively small change in diet can affect the insect’s feeding behavior [42,54,55]. Probably temperature like other environmental stresses affects feeding, as aphids at a higher metabolic level increase their feeding intensity, which in turn would be associated with increased activity of enzymes.

The effect of temperature on insect metabolism could depend on many factors. Studies on pupae of *Platynota stultana* confirmed the effect of temperature on the metabolic rate. The metabolic rate tripled when the temperature increased from 10 to 20 °C and doubled when the temperature rose from 20 to 30 °C [56,57]. It has been shown that pupae of *Musca domestica*, with an increase in temperature from 5 to 41 °C, increase their emission of heat and CO_2_ [49]. Similar correlations were observed in pupae of *Cydia pomonella*, where with the temperature increase, the heat emitted by pupae increased [58]. The metabolic reaction of insects to temperature increase may depend on the species [59]. Comparison of the metabolic activity of the Egyptian bee *Apis mellifera lamarcki* and the European *Apis mellifera carnica* at high temperatures showed small changes in heat emission in *A. mellifera lamarcki*, while showing a significant decrease in the metabolic rate in *A. mellifera carnica*. Studies of heat production in male and female *Galleria malonella* at temperatures of 20 °C and 30 °C have shown that heat emission can also be associated with gender of the insects [60]. Metabolic analyses have shown that high and low temperatures can inhibit *Leptocybe invasa* metabolism and shorten the lifespan of this species [61]. Similar relationships were observed when analyzing changes in heat flow in *Diaphorina citri* [62].

In light of observed global warming, our earlier results indicated that an increase in temperature negatively affected the biology of *A. pomi* [48]. Current studies, however, have shown that *A. pomi* exposed for a short time to high temperatures (up to 28 °C), protected itself against excessive ROS induction, thanks to the coordinated interaction of antioxidative, oxidoreductive, and detoxification enzymes, and an increase in metabolic rate. However, long-term persistent high temperature (2 weeks) caused comprehensive reactions between the host plant and aphid, which resulted in high enzyme activity. This suggests that global warming will negatively affect *A. pomi* defense responses to temperature stress. Previous studies have shown that global warming has had a positive effect on many insect species. Due to the increase in temperature, these species have been able to expand their range, develop more rapidly, increase the number of generations, and have higher overwintering survival—for example, in the case of butterflies or beetles [63,64,65,66,67]. For aphids, which are mainly associated with a temperate climate, prolonged temperature increase is not favorable, as confirmed by our research. For species developing at temperatures that go beyond the temperature optimum, temperature increase causes a negative impact on the development cycle and the number of generations during the growing season [4,68]. In general, higher temperatures are advantageous for aphids, enabling faster spring flights and colonization of new areas, but mostly as a cosmopolitan pest species (generalist) that originates in warmer climates [2]. The opposite situation takes place in indigenous aphid species inhabiting temperate climatic zones. For them, an increase in temperature may go beyond the optimum to which they are best adapted. Recent research suggested that the occurrence of the host plant does not necessarily correlate with climatic niche (especially for specialists) and that physiological limits can constrain aphid distribution [69]. On the other hand, higher temperatures favor parthenogenetic reproduction and the survival of active individuals throughout the year [2,70]. Climate warming also has a negative impact on predator species and parasites that are related to aphids [2]. Our studies have confirmed that temperature is a key factor that, by affecting physiological response and metabolism, can limit the development and distribution of aphids, as in the example of *A. pomi.*

## 5. Conclusions

In summary, our analyses indicate that the aphid’s response to temperature increase can be considered in two stages. The first stage of the aphid response includes metabolic changes and changes in the activity of antioxidant, detoxifying, and oxidoreductive enzymes in their tissues. Our analyses show that the increase in temperature causes higher activity of antioxidant, detoxification, and oxidoreductive enzymes, as well as an increase in the metabolic rate. Thanks to these adaptive mechanisms, the species protects itself against OS induction. The second stage response is caused by the influence of the host plant under long-term biotic and abiotic stress. Such stress could increase the defense mechanisms of the plant, which in turn intensifies the aphid defense response. *Aphis pomi* defense responses varied depending on temperature and were highest at 28 °C. Aphids are able to react quickly to environmental stress due to the flexible activity of enzymes, which ensure adaptation to the environment.
